# Development of the Parent Perceptions of Physical Activity Scale (PPPAS): Results from two studies with parents of infants and toddlers

**DOI:** 10.1371/journal.pone.0213570

**Published:** 2019-05-29

**Authors:** Kimberley D. Lakes, Jessica Vaughan, Shlomit Radom-Aizik, Candice Taylor Lucas, Annamarie Stehli, Dan Cooper

**Affiliations:** 1 Department of Psychiatry and Neuroscience, School of Medicine, University of California, Riverside, Riverside, California, United States of America; 2 Pediatric Exercise and Genomics Research Center (PERC), Department of Pediatrics, University of California, Irvine, Irvine, California, United States of America; 3 Children’s Hospital Los Angeles, Los Angeles, California, United States of America; Hong Kong Polytechnic University, HONG KONG

## Abstract

Physical activity (PA) is important from birth to promote health and motor development. Parents of young children are gatekeepers of opportunities for PA, yet little is known about their perceptions of PA. We describe the development of the Parent Perceptions of Physical Activity Scale (PPPAS) across two studies (*N* = 241 parents). In Study 1, 143 parents of infants and toddlers recruited from neonatal intensive care units (NICUs) and childcare centers completed a 48-item PPPAS. In Study 2, 98 parents of premature infants completed the revised 34-item PPPAS. Study 1 principal components analysis (PCA) identified three components (benefits of, barriers to, and perceived influence on PA), and the scale was reduced. Scores for Perceived Barriers to PA were significantly different between groups, *U* = 1,108, *z* = -4.777, *p* < .0001, with NICU parents reporting more barriers to PA than childcare parents. In Study 2, PCA revealed the same components, and the scale was further reduced to 25 items. Three subscales measuring perceived benefits of, barriers to, and influence over an infant’s PA produced Cronbach’s alphas of .93, .85, .81, respectively. Results demonstrated sufficient construct validity and internal consistency of PPPAS scores, supporting its use in future PA research.

## Introduction

National guidelines recommend that starting at birth, infants should engage in daily age appropriate physical activity [1]. Physical activity (PA) in infants (described as including exploratory movement, physical interactions with the environment, tummy time, active play, and opportunities to develop movement skills) has been associated with better overall health, development of motor skills, social skills, and maintenance of healthy weight [2]. Recent increases in health problems, including childhood obesity, highlight the importance of the need to actively promote PA as early as during infancy.

Research has suggested that parental beliefs about PA are directly related to child PA behaviors [3] and that the most effective PA interventions include a family component [4]. Questionnaires have been used to assess parental influence on the activity levels of their children [5]; children’s participation in organized sports, sedentary activities, and feeding behaviors [6]; psychosocial influences on children’s PA [7, 8]; and parental beliefs and attitudes about feeding and obesity [9]. These studies focused primarily on pre-school and school age children; little research has addressed parental influence on and perceptions of PA in infants. In spite of the important role caregivers play in promoting PA, a review of the current literature yielded no published instrument designed to assess caregiver attitudes towards current or future PA in preterm or term infants.

Additionally, it is likely that unique barriers exist in implementing early health (including PA) interventions in infants, especially in the preterm infant population. Prior research suggests that mothers of premature infants may perceive their infants as fragile, but still strong and capable of participating in infant PA [10]. Since participation in PA early in life requires parental support and active participation, a more in-depth understanding of parental perceptions of PA for their premature infants is vital for the success of this type of intervention, particularly to examine how perceived benefits of PA might be offset by perceived barriers to PA for children with health risks. We designed the current studies to evaluate a measure (Parent Perceptions of Physical Activity Scale: PPPAS) we developed to assess parent or caregiver perceptions of infant and toddler PA. Our research questions and hypotheses included:

1. Will pilot items load on predicted subscales? We predicted that the majority of items generated for the infant PPPAS would load on the predicted subscales and that we could reduce the data by eliminating some items to result in a scale of manageable length.
2. Will the PPPAS scales produce scores with sufficient internal consistency? We predicted that there would be sufficient internal consistency (alpha > .80). Ponterotto & Ruckdeschel [11] reported that an alpha of > .80 would be “good” for scales with between 7 and 11 items in a study with 100–300 participants.

## Study 1

### Methods

#### Participants and procedures

This study was approved by the University of California Irvine and Memorial Care Health System Institutional Review Boards. The pilot version of the PPPAS was distributed to mothers of infants hospitalized in several neonatal intensive care units (NICUs) in Southern California. Because this was our first PPPAS study, and we expected our sample would not be large enough to study potential differences between mothers and fathers, we limited recruitment in the first phase to mothers. Eligible mothers in the NICU were approached by study staff and asked to participate. The instrument also was distributed to mothers of infants ages 8–14 months at 17 local childcare facilities. Participants were asked to report racial/ethnic, socioeconomic, educational, and family information, but no participant identifiers were obtained. A drop-off box was available at the childcare centers’ front desks in order for parents to submit the questionnaire anonymously. Participants were given the option of completing the survey in either English or Spanish, were informed that their participation was voluntary and anonymous, and were given $5 compensation at the time of survey distribution. 143 participants (Mean age 31.4 years, standard deviation 5.3) were included in Study 1. Table 1 reports the demographic characteristics of study participants and their children.

**Table 1:** Demographic characteristics of study 1 parent participants and their infants.

	N (%)
**Survey Language**	
English	120 (86%)
Spanish	20 (14%)
**Collection Site**	
Childcare Facility	75 (52.4%)
Neonatal Intensive Care Unit	68 (47.6%)
**Parent’s BMI**	
Obese (30.0 and Above)	24 (18%)
Overweight (25.0–29.9)	31 (23%)
Healthy Weight (18.5–24.9)	76 (57%)
Underweight (Below 18.5)	3 (2.2%)
**Race** ^**a**^	
Hispanic or Latina	61
Black or African American	6
Asian	27
White	49
American Indian/Alaska Native	0
Pacific Islander	2
Other	2
**Marital Status**	
Single Never Married	14 (99%)
Married	102 (71.8%)
Divorced	3 (2.1%)
Separated	3 (2.1%)
Living with a partner/not married	20 (14.1%)
**Education**	
Did not complete High School	15 (10.6%)
High School Diploma/GED	26 (18.3%)
Some College/Vocational School	32 (22.5%)
Bachelor’s Degree	34 (23.9%)
Advanced Degree	35 (24.6%)
**Annual Household Income ($)**	
Less than $1,000	19 (14%)
$1,000- $2,999	31 (22.8%)
$3,000- $4,999	15 (11%)
$5,000- $6,999	19 (14%)
$7,000- $8,999	11 (8.1%)
$9,000- $10,999	8 (5.9%)
$11,000- $12,999	2 (1.5%)
$13,000 or more	31 (22.8%)
**Number of Children**	
1	58 (40.8%)
2	58 (40.8%)
3	15 (10.6%)
4	10 (7.0%)
5	1 (0.7%)
**Infant’s Age**	
0 to <2 months	47 (34.1%)
2 to <4 months	11 (7.9%)
4 to <6 months	2 (1.4%)
6 to <8 months	10 (7.2%)
8 to <10 months	20 (14.5%)
10 to <12 months	26 (18.8%)
12 to < 14 months	15 (10.8%)
14 months and above	8 (5.2%)
**Infant’s Gender** ^**b**^	
Male	81 (52.3%)
Female	74 (47.7%)
**Was infant premature?**	
Yes	36.4%
No	62.2%

Note.

^a^ Some participants reported more than one race.

^b^ Parents of multiples were included.

#### Parent Perceptions of Physical Activity Scale (PPPAS)

To develop the initial content for the PPPAS, qualitative, semi-structured, open-ended interviews were conducted with 23 mothers of preterm infants to explore caregiver perceptions of PA in infants and potential barriers to promoting PA for babies. We focused on parents of preterm infants because our earlier work with them indicated that they perceived their children as especially fragile and had concerns about allowing them to be active. This led to research questions about parent perceptions about potential benefits of and barriers to PA. Enrolled mothers either had an infant currently admitted in the NICU [10] or were participating in a three-week daily caregiver assisted exercise program [12]. The following conceptual categories emerged from the interviews and guided the development of the questionnaire:

1. Caregivers’ perceptions of their roles in promoting their infant’s health (role)
2. Perceived benefits of infant physical activity or exercise (benefit)
3. Perceived barriers to encouraging or promoting infant physical activity (barrier)
4. Caregivers’ fears related to the perceived fragility of their infants (fear).

The 48 pilot items for the Perceptions of Pediatric Activity Scale (PPAS), contained four categories of questions, as described above, with 5, 27, 4, and 12 items, respectively. For a more detailed explanation of the methods used to transform this qualitative data into a quantitative survey tool, see Olshansky et al. [13].

Piloted PPPAS items are listed in Table 2. The following are example items: “My infant will live longer if I encourage him/her to be an active baby,” for the category “role”; “Physical activity increases my infant’s muscle strength”, and”Physical activity now will keep my infant from having weight problems in the future,” for the category “benefit”; “Encouraging infants to do physical activity takes too much time,” for the category “barrier”; and “I am scared that physical activity will be harmful for my baby”, and “My baby will not be strong enough for physical activity in childhood,” for the category “fear”.

**Table 2:** Study 1 principal components analysis.

Items	Rotated Component Coefficients			Communalities
	1	2	3	
***Benefits of Physical Activity (PA)***				
PA allows my infant to carry out normal activities without becoming tired.	**.785**	-.075	.259	.690
Activity improves functioning of my infant’s cardiovascular system.	**.724**	-.093	.144	.554
My infant will live longer if I encourage him/her to be an active baby.	**.713**	.097	.097	.535
My infant’s physical endurance is improved by encouraging him/her to be active.	**.690**	.015	.015	.574
PA improves my infant’s flexibility.	**.683**	.002	.002	.588
PA increases my infant’s mental alertness.	**.670**	-.044	-.044	.537
PA increases my infant’s stamina.^a^	**.645**	-.025	-.025	.478
Encouraging my infant to be active will let me have contact with my infant.	**.636**	-.143	-.143	.442
Increasing activity increases my infant’s level of physical fitness.	**.636**	-.431	-.431	.616
PA now will keep my infant from having weight problems in the future.	**.633**	-.204	-.204	.459
My infant has improved feelings of well being from PA.	**.615**	-.359	-.359	.516
Exercising helps my infant sleep better at night.	**.598**	-.029	-.029	.430
My infant’s disposition is improved by PA.^a^	**.597**	-.056	-.056	.494
My infant’s muscle tone is improved with PA.^a^	**.576**	-.430	-.430	.517
PA improves overall body functioning for my infant.	**.557**	-.204	-.204	.547
PA makes my infant feel relaxed.^a^	**.556**	.040	.040	.312
PA gives my infant a sense of personal accomplishment.	**.525**	-.537	-.014	.565
PA is good entertainment for my infant.	**.513**	-.193	-.193	.477
PA improves my infant’s mental health.	**.509**	-.217	-.217	.368
PA helps my infant decrease fatigue.^a^	**.504**	.172	.172	.298
PA decreases feelings of stress and tension for my infant.^a^	**.437**	-.459	-.210	.445
My infant feels proud when doing PA.	**.420**	-.116	-.116	.355
I will improve future health by encouraging PA in my infant.	**.414**	-.256	-.256	.381
PA increases my infant’s muscle strength.	**.410**	-.579	.114	.517
Increased PA during infancy (up to 12 months) is a sign that a child will be a more active during elementary school.^a^	**.312**	.233	.233	.200
***Perceptions of Barriers to PA***				
My family members do not encourage me to do PA with my infant.	-.101	**.767**	-.150	.621
My spouse (or significant other) does not encourage infant activity.	-.094	**.751**	-.124	.589
Encouraging infants to do PA takes too much time.	-.017	**.750**	-.119	.576
I am scared that PA will be harmful for my baby.	-.056	**.724**	-.299	.616
My infant’s physical endurance is improved by encouraging him/her to be active.	-.036	**.655**	-.220	.478
Encouraging my infant to be active will take too much time from my family responsibilities.	-.029	**.647**	-.065	.423
I should try to decrease PA in my baby.	-.115	**.644**	-.100	.438
PA improves my infant’s flexibility.	.049	**.499**	-.018	.252
PA in infancy will make my baby sicker.	-.063	**.465**	-.535	.507
It is dangerous for my baby to be physically active.	-.069	**.397**	-.609	.533
I am worried about my baby’s health.^a^	.043	**.382**	-.281	.226
My baby is not strong enough for PA.^a^	.008	**.342**	-.604	.482
I am scared I will hurt my baby.	.141	.274	-.568	.418
***Perceptions of Caregiver Influence on PA***				
I feel that PA will be important for my child in elementary school.^a^	.384	-.116	**.626**	.553
I plan to encourage PA when my baby is in elementary school.	.273	-.137	**.587**	.438
PA in childhood will make my child healthier.	.335	-.131	**.584**	.470
My infant enjoys PA.	.151	-.439	**.564**	.534
My patterns of PA will strongly impact the patterns of PA that my child will develop over the course of his/her life.	.228	.048	**.521**	.325
My attitudes about exercise will strongly impact my child’s attitude towards exercise over the course of his/her life.	.320	.102	**.506**	.369
My baby needs to rest in order to grow.^a^	.188	.017	.265	.106

^a^ Items removed after Study 1

### Analyses

The following numerical values were assigned to the survey responses: Strongly Agree = 1, Agree = 2, Disagree = 3, and Strongly Disagree = 4. SPSS 23 was used to conduct all analyses. First, we conducted principal components analysis (PCA) to examine construct validity and to identify items that could be eliminated in an effort to create a scale of manageable length. In addition, we evaluated the internal consistency of each of the PPPAS scales generated by PCA using Cronbach’s alpha and the distributions using the Shapiro-Wilk test of normality.

### Results

#### Principal components analysis (PCA)

Prior to analysis, we assessed the appropriateness of our data for PCA. After inspection of the correlation matrix, it was determined that three items failed to produce correlation coefficients greater than 0.3. We removed these items from further analysis. The remaining 43 items were deemed appropriate for PCA, based on an overall Kaiser-Meyer-Olkin (KMO) measure of .785 and a significant (*p* < .0001) Bartlett’s test of sphericity [14–16].

PCA revealed three primary components that had eigenvalues greater than 1; the three components explained 29.11%, 10.94%, and 6.28% of the variance, respectively, and visual inspection of the scree plot indicated that three components should be retained [17]. Moreover, a three-component solution met the interpretability criterion. The three-component solution explained 46.33% of the total variance. We used a Varimax orthogonal rotation to help with interpretability. The rotated solution exhibited “simple structure” [18]. Results are presented in Table 2, with notations indicating which items were removed prior to subsequent analyses (limited to a reduced 34-item scale).

Items were removed before the next set of analyses as noted in Table 2. Identification of items to delete was based on several criteria; if an item loaded on more than one factor, included words that participant feedback suggested were too technical (e.g., “stamina”), had substantial overlap in content with another item (e.g., added no new information and increased respondent burden), or loaded weakly or not at all, we deleted the item. We subsequently confirmed the structure of the revised 34-item scale by conducting a second PCA with factors fixed to three. The items were appropriate for PCA as evidenced by the correlation matrix, a KMO of .835, and a significant (*p* < .0001) Bartlett’s test of sphericity [14–16]. The three factors explained 31.40%, 12.15%, and 6.28% of the variance, for a total of 48.83% of the variance. The rotated solution supported prior results indicating the scale had a “simple structure” [18]. Thus, we progressed to the next analyses using this 34-item infant PPPAS.

#### Reliability: Internal consistency coefficients

Cronbach’s alpha was computed for all three subscales of the reduced 34 –item PPPAS and yielded coefficients of .92, .87 and.74 for the Benefits of PA, Perceptions of Barriers to PA, and Perceptions of Parental Influence on PA subscales, respectively.

#### Measures of central tendency and distribution

Table 3 reports descriptive findings for the revised subscales. Shapiro-Wilk tests for the three scales indicated that data were not normally distributed, and means indicated that parents tended to agree with statements regarding perceived benefits of PA and tended to disagree with potential barriers to PA.

**Table 3:** Descriptive Statistics and Internal Consistency Coefficients for 30-item Infant and Toddler PPPAS (Study 1).

	Mean (SD)	Skewness	Kurtosis	Shapiro-Wilk	*p*
Beliefs in the Benefits of PA	1.68	-.177	-.781	.949	.000
Perceptions of Barriers to PA	3.42	-.89	1.09	.927	.000
Perceived Influence on PA	1.43	.876	.526	.901	.000

#### Group comparisons

As we hypothesized that parents of infants with medical concerns may be more likely to perceive barriers to PA, we also analyzed group differences. We conducted Mann-Whitney *U* tests to determine if there were differences in perceived benefits of, barriers to, and influence on PA between parents of infants in the NICU and parents of infants in childcare centers (a slightly older and healthier population). Median scores for Perceived Benefits of PA were not significantly different between parents of NICU babies (1.81) and parents of babies in childcare centers (1.78), *U* = 1,908, *z* = 0.370, *p* = .711. Median scores for Perceived Barriers to PA were significantly different between parents of NICU babies (3.73) and parents of babies in childcare centers (3.27), *U* = 1,108, *z* = -4.777, *p* < .0001. The distribution of scores for Perceived Influence on PA was also significantly different, *U* = 2,429, *z* = 2.177, *p* = .029, though the medians were identical (1.40).

### Implications for future development of the PPPAS

Based on results from this study, 34 items were retained: 18 Benefits of Physical Activity items, 11 Barriers to Physical Activity items, and 5 Impact of Caregiver Influence items. Rather than eliminating all items that didn’t load strongly, in some cases the wording was changed in order to try to improve interpretability. In addition, two new items were generated for the caregiver influence scale and included in the scale for future research; these items were included in efforts to strengthen Cronbach’s alpha for this third scale. Moreover, results suggested that although most parents of infants perceive PA as beneficial, parents of premature or otherwise medically fragile infants may perceive more barriers to promoting PA in their infants and may perceive themselves as having less influence over their child’s level of PA. Thus, in the next stage of this research, it seemed prudent to further study the scale in a population where parents’ perceived barriers and influence were relevant factors.

## Study 2

### Methods

#### Participants and procedures

This study also was approved by the University of California Irvine Institutional Review Board. The revised version of the PPPAS was administered to a group of mothers enrolled in a study assessing the effects on body composition of a yearlong PA intervention for preterm infants. Preterm infants were enrolled in the study before discharge from the NICU and were followed until one year after discharge. Participants were randomly assigned to one of two groups: either an “Exercise” group in which caregivers perform daily exercises with their infants or a “Control” group in which caregivers participate in daily structured social activities with their infants. All caregivers enrolled in the study were asked to complete the revised PPPAS at the time of enrollment. Caregivers (mean age 30.61 years) of 98 premature infants who completed at least 32 items of the PPPAS were included in Study 2 (several caregivers left multiple items blank on the PPPAS, and, for this study, we required completion of at least 32 of 36 items in order to reduce effects of missing data). Table 4 reports the demographic characteristics of the parent study participants and their infants. The study recruited primary caregivers as they would be the ones trained to implement the home intervention program; although we did not restrict recruitment to mothers, the sample consisted of mothers as in these families, they were identified as the primary caregivers for the infants.

**Table 4:** Demographic characteristics of 98 parent participants and their premature infants (Study 2).

	N (%)
**Race**	
Hispanic or Latina	66 (69%)
Black or African American	10 (10%)
Asian	7 (7%)
White, Non Hispanic	25 (26%)
American Indian/Alaska Native	1 (1%)
Pacific Islander	3 (3%)
Other/Mixed Ethnicities	38 (39%)
Declined to State	14 (14%)
**Marital Status**	
Single Never Married	13 (13%)
Married	57 (59%)
Divorced	0
Separated	2 (2%)
Living with a partner/not married	25 (26%)
**Education**	
Did not complete High School	12 (12%)
High School Diploma/GED	26 (27%)
Some College/Vocational School	31 (32%)
Bachelor’s Degree	15 (16%)
Advanced Degree	13 (13%)
**Monthly Household Income ($)**	
Less than $1,000	20 (21%)
$1,000- $2,999	33 (35%)
$3,000- $4,999	14 (15%)
$5,000- $6,999	8 (8%)
$7,000- $8,999	6 (6%)
$9,000- $10,999	1 (1%)
$11,000- $12,999	1 (1%)
$13,000 or more	12 (13%)
**Number of Children**	
1	45 (48%)
2	22 (24%)
3	14 (15%)
4	9 (10%)
5	3 (3%)
**Infant’s Gender**	
Male	45 (46%)
Female	53 (54%)

### Analyses

SPSS 23 was used to conduct all analyses. We used principal components analysis (PCA) to examine the structure of the scale, and we evaluated the internal consistency of each of the PPPAS scales (revised based on PCA results) using Cronbach’s alpha and the distributions of using the Shapiro-Wilk test of normality.

### Results

#### Principal components analysis

We ran a PCA on a revised 36-item PPPAS (the retained 34 items plus 2 new items generated for inclusion in study 2). Prior to analysis, we assessed the suitability of PCA for our data. Upon inspection of the correlation matrix it was determined that all variables except one had at least one correlation coefficient greater than 0.3. The item that was not correlated with the others was removed from analyses, resulting in analysis of 35 items. The overall Kaiser-Meyer-Olkin (KMO) measure was 0.78. Bartlett’s test of sphericity was statistically significant (*p* < .0005), indicating that the data was likely factorizable [14–16].

PCA revealed three primary components that had eigenvalues greater than 1; the three components explained 31.31%, 17.24%, and 6.23% of the variance, respectively. A visual inspection of the scree plot indicated that three components should be retained [17]. Moreover, a three-component solution met the interpretability criterion. The three-component solution explained 54.79% of the total variance. We used a Varimax orthogonal rotation to help with interpretability, and the rotated solution exhibited “simple structure” [18]. Interpretation of the data was consistent with the categories the questionnaire was designed to measure, with strong loadings of opinions regarding the benefit of PA on Component 1, beliefs related to perceived barriers to PA on Component 2, and perceptions of parental influence on child’s PA loading on Component 3 (See Table 5). Several items either loaded strongly onto both components 1 and 3, with the stronger coefficients not falling within the hypothesized category, or they loaded onto a scale other than the one for which they were designed. Thus, these four items were removed from the final scale. Several additional items (notated in Table 5) were also removed as they were redundant, and one of our goals was to generate a brief scale sensitive to participant time constraints. The three components (containing 25 retained items) were used to create three PPPAS subscales: Benefits of PA (16 items), Perceptions of Barriers to PA (7 items), and Perceptions of Caregiver Influence on PA (2 items).

**Table 5:** Study 2 Principal components analysis.

Items	Rotated Component Coefficients			Communalities
	1	2	3	
***Benefits of Physical Activity (PA)***				
My infant has improved feelings of well being from PA.	**.833**	-.015	-.139	.713
My infant feels proud when doing PA.^b^	**.827**	-.058	-.131	.704
PA improves my infant’s mental health.^b^	**.798**	.109	-.021	.649
PA is good entertainment for my infant.	**.768**	-.010	.372	.728
Encouraging my infant to be active will let me have contact with my infant.	**.764**	.049	.267	.657
PA gives my infant a sense of personal accomplishment.	**.717**	-.055	-.023	.518
My infant’s physical endurance is improved by encouraging him/her to be active.	**.700**	.036	.371	.629
PA increases my infant’s muscle strength.	**.691**	.081	.095	.493
Activity improves functioning of my infant’s cardiovascular system.	**.643**	-.019	.322	.517
Increasing activity increases my infant’s level of physical fitness.	**.638**	-.014	.404	.570
PA improves overall body functioning for my infant.	**.622**	.245	.391	.601
PA increases my infant’s mental alertness.	**.616**	-.007	.444	.576
PA allows my infant to carry out normal activities without becoming tired.	**.600**	.229	.128	.429
PA now will keep my infant from having weight problems in the future.	**.594**	-.071	.294	.444
My infant enjoys PA.	**.552**	.080	-.064	.315
Exercising helps my infant sleep better at night.^b^	**.452**	.031	**.598**	.563
PA in childhood will make my child healthier.	**.542**	.256	.438	.551
PA improves my infant’s flexibility.	**.537**	.191	.498	.574
My infant will live longer if I encourage him/her to be an active baby.	**.469**	-.051	.466	.440
***Perceptions of Barriers to PA***				
PA in infancy will make my baby sicker.^b^	.085	**.843**	.047	.721
I am scared that PA will be harmful for my baby.	.037	**.819**	.144	.693
It is dangerous for my baby to be physically active.^b^	-.019	**.808**	.132	.671
Encouraging my infant to be active will take too much time from my family responsibilities.	.083	**.790**	-.209	.675
My family members do not encourage me to do PA with my infant.	.070	**.788**	-.265	.697
I should try to decrease PA in my baby.	.040	**.764**	.211	.631
My spouse (or significant other) does not encourage infant activity.^b^	-.116	**.706**	.051	.514
I am scared I will hurt my baby.^b^	.057	**.670**	.262	.521
Encouraging infants to do PA takes too much time.	.092	**.665**	-.204	.493
My baby will not be strong enough for PA in childhood.	-.145	**.653**	.244	.507
PA is hard work for my infant.	.090	**.501**	-.165	.287
***Perceptions of Caregiver Influence on PA***				
My attitudes about exercise will strongly impact my child’s attitude towards exercise over the course of his/her life.	.056	.042	**.710**	.509
My exercise habits will strongly impact the exercise habits that my child will develop over the course of his/her life	.165	-.007	**.629**	.423
My child will learn exercise habits through watching my example.^a^^,^ ^b^	**.526**	-.065	**.199**	.321
I will improve future health by encouraging PA in my infant.^b^	**.530**	-.038	**.282**	.362
How much I value exercise will impact how active my child is.^a^^,^ ^b^	**.549**	.000	**.424**	.481

Note.

^a ^New items generated after Study 1, administered in Study 2.

^b^ Items removed after PCA for Study 2.

**Fig 1:**
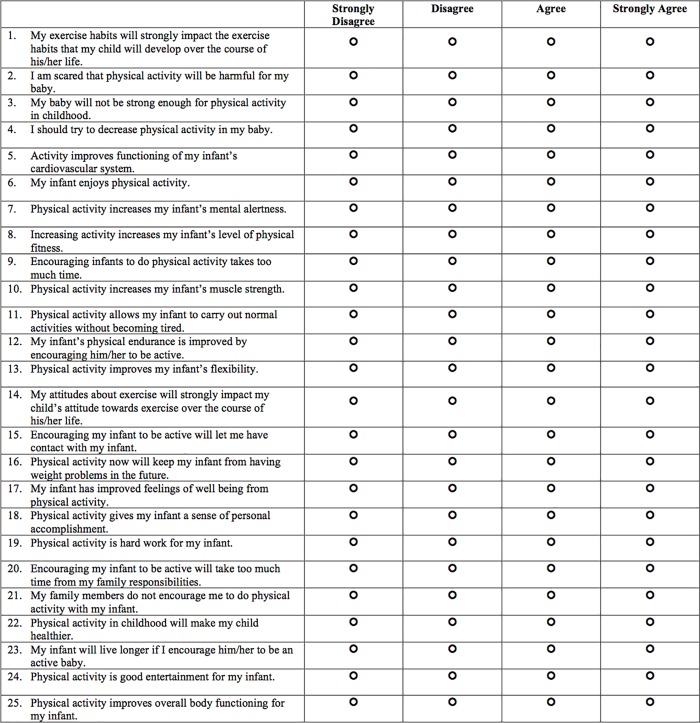
The PPPAS-Infant and toddler version. *Note*. Average items 5, 6, 7, 8, 10, 11, 12, 13, 15, 16, 17, 18, 22, 23, 24, 25 for the Benefits subscale; items 2, 3, 4, 9, 19, 20, 21 for the Barriers subscale; and items 1 and 14 for the Parental Influence subscale.

Next, we conducted a new PCA with the retained 25 items. All items were correlated, the KMO measure was .825, and Bartlett’s test of sphericity was statistically significant (*p* < .0001), indicating that the data was likely factorizable [14–16]. PCA results confirmed the three-factor solution. The three components explained 34.43%, 15.21%, and 6.84% of the variance, for a total of 56.48% variance explained. The scree plot and interpretability criteria both supported retention of three components. Finally, the rotated component matrix indicated that the items loaded on the hypothesized scales (see Table 6). Thus, we present a final 25-item PPPAS for infants (Fig 1).

**Table 6:** Principal components analysis for the final 25-item PPPAS.

Items	Rotated Component Coefficients			Communalities
	1	2	3	
***Benefits of Physical Activity (PA)***				
My infant has improved feelings of well being from PA.	**.755**	.025	-.261	.638
PA is good entertainment for my infant.	**.844**	.000	.189	.748
Encouraging my infant to be active will let me have contact with my infant.	**.782**	.043	.113	.626
PA gives my infant a sense of personal accomplishment.	**.695**	-.067	-.182	.520
My infant’s physical endurance is improved by encouraging him/her to be active.	**.792**	.063	.180	.663
PA increases my infant’s muscle strength.	**.662**	.065	.053	.445
Activity improves functioning of my infant’s cardiovascular system.	**.751**	-.052	.088	.575
Increasing activity increases my infant’s level of physical fitness.	**.763**	-.055	.213	.630
PA improves overall body functioning for my infant.	**.755**	.240	.202	.669
PA increases my infant’s mental alertness.	**.675**	-.009	.327	.562
PA allows my infant to carry out normal activities without becoming tired.	**.602**	.264	.099	.442
PA now will keep my infant from having weight problems in the future.	**.594**	-.092	.059	.365
My infant enjoys PA.	**.538**	.114	-.129	.319
PA in childhood will make my child healthier.	**.697**	.224	.215	.582
PA improves my infant’s flexibility.	**.661**	.203	.386	.626
My infant will live longer if I encourage him/her to be an active baby.	**.629**	-.072	.228	.453
***Perceptions of Barriers to PA***				
I am scared that PA will be harmful for my baby.	.087	**.768**	.182	.631
Encouraging my infant to be active will take too much time from my family responsibilities.	.041	**.830**	-.116	.705
My family members do not encourage me to do PA with my infant.	-.007	**.817**	-.184	.701
I should try to decrease PA in my baby.	.148	**.736**	.205	.606
Encouraging infants to do PA takes too much time.	.011	**.710**	-.121	.519
My baby will not be strong enough for PA in childhood.	-.108	**.607**	.346	.499
PA is hard work for my infant.	.066	**.560**	-.060	.321
***Perceptions of Caregiver Influence on PA***				
My attitudes about exercise will strongly impact my child’s attitude towards exercise over the course of his/her life.	.162	.008	**.796**	.659
My exercise habits will strongly impact the exercise habits that my child will develop over the course of his/her life. (1)	.242	-.049	**.744**	.614

#### Reliability: Internal consistency coefficients

Cronbach’s alpha was computed for all three subscales of the PPPAS and yielded coefficients of .94, .85, and .81 for the Benefits of PA, Perceptions of Barriers to PA, and Perceptions of Caregiver Influence on PA subscales, respectively.

#### Measures of central tendency and distribution

Descriptive statistics and measures of central tendency and distribution are presented in Table 7. The Shapiro-Wilk tests for all three subscales suggested that data were not normally distributed. Means for the three subscales indicated that parents tended to endorse beliefs in the benefits of PA and their ability to influence PA. Their endorsement of a lack of barriers to PA was somewhat weaker.

**Table 7:** Descriptive statistics for final 25-item infant PPPAS.

	Mean (SD)	Skewness	Kurtosis	Shapiro-Wilk	*p*
Beliefs in the Benefits of PA	3.46 (0.39)	-0.065	1.379	.910	< .0001
Perceptions of Barriers to PA^a^	3.08 (0.57)	-1.169	2.795	901	< .0001
Perceived Influence on PA	3.44 (0.63)	-1.118	1.535	.799	< .0001

Note

^a^Reverse-scored so that higher scores reflect fewer perceived barriers.

## Discussion

Current guidelines support the longitudinal benefits of encouraging PA early in life. The National Association for Sport and Physical Education [19] recognized the importance of PA early in life and presented specific guidelines for children from birth to 5 years of age. In infants from birth to 12 months PA recommendations do not specify duration but include promoting the exploration of the environment, promoting motor skill development and not restricting movement for prolonged periods of time. In toddlers, it is recommended that they participate in at least 30 minutes of structured and 60 minutes of unstructured PA daily. Similarly in *Caring for our Children*, a collection of national standards written by American Academy of Pediatrics, American Public Health Association and National Resource Center for Health and Safety in Child Care and Early Education [20] it is recommended that infants birth to 12 months should be taken outside two to three times per day for outdoor play.

Parents or caregivers play a key role in the promotion of PA, particularly among infants and toddlers. The PPPAS was designed to provide a tool that professionals in various fields (e.g., pediatrics, early childhood intervention, PA and motor development research) can use to measure parent perceptions of PA in order to identify potential issues that might impact parent support for and promotion of PA. Our results yielded a 25-item scale (PPPAS), with three subscales: Beliefs in the Benefits of PA, Perceptions of Barriers to PA, and Perceptions of Caregiver Influence on PA. In the two studies presented, the PPPAS subscales yielded strong internal consistency reliability scores, and factors were consistent with the hypothesized conceptual categories, suggesting that the PPPAS is a useful research tool and potentially a promising screening tool.

Utilizing the PPPAS as a screening tool may assist primary care providers in engaging parents of young children in primary prevention of obesity and other health consequences associated with sedentary lifestyles. The PPPAS may be useful in predicting parents’ willingness to implement infant PA interventions as well as to identify any barriers or fears that might impact compliance with PA recommendations. For example, as we illustrated in Study 1, parents of infants in the NICU (i.e., a population with special health care needs) may perceive more barriers to their child’s ability to engage in PA and may see their ability to influence this involvement as weaker. Education around the importance of PA and appropriate PA for children with disabilities or medical concerns could reduce these perceived barriers, possibly increasing opportunities for PA and motor development. Thus, the PPPAS may help primary care providers individualize the anticipatory guidance they provide to parents of infants.

Moreover, the PPPAS can be used as a research tool to study caregiver or parent perceptions of PA in a variety of infant populations. It also could be used to evaluate change over time in parent perceptions; for example, the PPPAS could be used as an outcome measure to assess change in perceptions following a PA intervention or a parent education intervention. Lastly, researchers examining a variety of social and environmental factors that influence infant health could include the PPPAS in their battery of assessments.

### Limitations and the need for future research

Study limitations include the characteristics of the samples studied, and, as is the case with most instruments, results cannot be generalized to all populations. Researchers using the PPPAS with other infant and toddler samples should independently evaluate the reliability of scores derived in those studies. Further psychometric analysis of the PPPAS is warranted as well and should include studies examining the test-retest reliability, sensitivity, and specificity of scores. Further research should be conducted with fathers in order to compare fathers’ perceptions with mothers’. Moreover, the content areas of the PPPAS were derived from prior research with fragile infant and toddler populations; thus, there may be additional content areas that would be useful to study that are not currently addressed by the PPPAS. Moreover, as with any questionnaire, respondents’ interpretations of items will be affected by their experiences, education, lifestyle and other individual and cultural factors; not all parents will define PA in the same way. Thus, this measure should be just one of multiple tools used in PA research with children; objective measurement of levels of PA and descriptive measures of types of PA are both important to consider alongside parent perceptions of PA.

Future research is needed to further address the validity of scores derived using the PPPAS–for example, do PPPAS scores predict parent behaviors related to PA? Do PPPAS scores predict actual levels of infant and toddler PA? Do they predict motor development outcomes in young children? What other factors interact with parental beliefs to influence an infant’s or toddler’s engagement in PA? How do the beliefs of other early caregivers (e.g., nannies, grandparents, daycare providers) impact early opportunities for PA? Future research should address how parental perceptions of PA affect infant health and development, particularly the risk for diseases associated with sedentary behavior. The PPPAS provides one potential tool for this type of research.
